# Prevalence and Latent Profiles of Cognitive Impairment in Patients with Heart Failure: A Secondary Data Analysis

**DOI:** 10.3390/jcm15155888

**Published:** 2026-07-28

**Authors:** Sun Hyoung Bae, Jinsun Park, Joonhan Shin

**Affiliations:** 1 College of Nursing, Research Institute of Nursing Science, Ajou University, Suwon 16499, Republic of Korea; 2 Department of Cardiology, School of Medicine, Ajou University, Suwon 16499, Republic of Korea

**Keywords:** heart failure, cognitive dysfunction, latent profile analysis, neuropsychological tests, anxiety, depression

## Abstract

**Background/Objectives:** Cognitive impairment can weaken self-management competency in heart failure (HF) patients. This study estimated domain-specific cognitive impairment prevalence using actuarial criteria and identified latent cognitive profiles among Korean HF outpatients. **Methods:** Using a previously collected cross-sectional dataset of 106 HF outpatients, cognitive function was assessed with the Seoul Neuropsychological Screening Battery-II. Actuarial criteria determined domain-specific impairment, and latent profile analysis (LPA) derived cognitive profiles based on domain composite z-scores. **Results:** Any cognitive impairment (≥1 impaired domain) was present in 32.1% (95% confidence interval [CI], 24.0–41.5%), most frequently affecting executive function (17.9%) and memory (15.1%). LPA identified three exploratory profiles: relatively preserved (30.2%), memory-vulnerable (45.3%), and globally vulnerable (24.5%). In exploratory models, anxiety symptoms were associated with higher odds of memory-vulnerable profile membership (odds ratio [OR] = 2.81, 95% CI, 1.03–7.64) and overall vulnerability in a Firth penalized analysis (OR = 3.39, 95% CI, 1.46–8.11), whereas depressive symptoms showed no significant association. No associations remained statistically significant after false discovery rate correction. **Conclusions:** Cognitive vulnerability in HF outpatients is phenotypically heterogeneous and showed a possible, unconfirmed association with anxiety symptoms that did not remain statistically significant after correction for multiple comparisons. Outpatient HF care planning may benefit from considering both domain-specific deficits and latent cognitive profile patterns; however, these exploratory findings require validation in larger cohorts.

## 1. Introduction

Heart failure (HF) is a chronic disease with a growing global burden and substantial mortality, characterized by frequent readmissions arising from complex symptom, medication, and lifestyle management demands [1,2,3,4]. This highlights the need to systematically evaluate the factors that impede self-management competency in the outpatient setting.

Cognitive impairment is a key factor that can undermine self-management in patients with HF [5,6]. Cognitive impairment can delay the recognition of symptom deterioration, reduce treatment adherence, and increase readmission and mortality risk [7,8,9,10]. Thus, accurately characterizing the extent and pattern of cognitive impairment in patients with HF is important for improving clinical assessment and support. However, previous studies have reported a wide range in the prevalence of cognitive impairment among patients with HF, ranging from 30% to 80% [5,6]. These differences may reflect variation in participant characteristics, including inpatient status and disease severity, as well as differences in the cognitive assessment instruments and classification approaches used across studies [5,11]. Although brief screening tests are useful for rapidly detecting possible cognitive impairment in clinical practice, they may have limited ability to characterize vulnerabilities across specific cognitive domains [11,12].

The relationship between HF and cognitive impairment is likely to be heterogeneous, reflecting multiple overlapping mechanisms [8,13]. Consistent with this, previous studies have reported impairments in attention, memory, psychomotor speed, and executive function in patients with HF, although the severity and combination of affected domains have varied across studies and sample characteristics [10,14,15,16]. These findings suggest that cognitive impairment in HF may not represent a single uniform condition, but rather heterogeneous patterns of cognitive vulnerability. In this context, data-driven approaches such as latent profile analysis (LPA) may help identify distinct subgroups of patients based on patterns of domain-level cognitive performance [16,17,18]; in the present study, these domain-level patterns are referred to as cognitive vulnerability profiles.

Nevertheless, research using standardized neuropsychological testing to examine domain-specific cognitive function in Korean patients with HF remains limited. In particular, evidence is insufficient regarding cognitive impairment across domains and the extent to which distinct cognitive profiles are associated with sociodemographic and clinical characteristics in this population [19,20,21]. Because non-systematic cognitive assessment may limit appropriate clinical assessment and support, a more structured evaluation of cognitive vulnerability may be helpful in outpatient HF care [22]. Therefore, utilizing a previously established clinical dataset, the primary aim of this secondary analysis was to assess domain-specific cognitive function and estimate the prevalence of cognitive impairment in outpatients with HF using a standardized neuropsychological battery. As a second, exploratory aim, we sought to identify latent cognitive profiles based on domain-level z-scores and to preliminarily explore, as a hypothesis-generating analysis, their possible associations with sociodemographic and clinical characteristics.

## 2. Materials and Methods

### 2.1. Study Design

This study is a secondary analysis of cross-sectional data originally collected to evaluate psychosocial and clinical factors associated with self-management in outpatients with HF. The reporting of this study follows the Strengthening the Reporting of Observational Studies in Epidemiology (STROBE) guidelines.

### 2.2. Participants

#### 2.2.1. Setting and Participants

The primary dataset was derived from patients recruited from the cardiology outpatient clinic of a tertiary teaching hospital in Gyeonggi-do, Republic of Korea. During the initial study period, 116 consecutive patients diagnosed with heart failure by the attending cardiologist were recruited from among eligible outpatients attending the clinic. After eligibility review and exclusion of those with incomplete questionnaire or neuropsychological test data, 10 participants were excluded, resulting in a final analytic sample of 106 patients. Inclusion criteria were: (1) age ≥ 45 years; (2) a diagnosis of heart failure established by the attending cardiologist and present for ≥6 months; (3) hemodynamic stability; and (4) ability to communicate and complete questionnaires and neuropsychological testing. Exclusion criteria were: (1) history of heart transplantation; (2) transient HF attributable to reversible precipitants (e.g., sepsis, anemia, or hyperthyroidism); (3) acute cardiovascular conditions (e.g., acute aortic dissection or hypertensive crisis); (4) history of conditions with a major direct impact on cognition (e.g., stroke or dementia); and (5) use of central nervous system-acting medications that could substantially affect cognitive testing (e.g., sedatives, antipsychotics, or other agents with significant cognitive side effects).

#### 2.2.2. Sample Size Considerations

Because this study is a secondary analysis, the sample size was predetermined by the available dataset (*N* = 106). However, to assess whether the sample provided adequate precision for the primary aim of estimating cognitive impairment prevalence, we applied the standard formula for a single proportion in cross-sectional studies [23]. Based on prior meta-analytic estimates suggesting a prevalence of cognitive impairment of approximately 40–41% in HF [24], assuming *p* = 0.41, a 95% confidence level (*Z* = 1.96), and an absolute precision of *d* = 0.10, the minimum required sample size was approximately 93. Accordingly, the present sample (*N* = 106) was considered to provide reasonable precision for estimating prevalence.

### 2.3. Measures

#### 2.3.1. Cognitive Function

Cognitive function was assessed using the Seoul Neuropsychological Screening Battery-II (SNSB-II), a standardized neuropsychological battery validated in Korean populations with age- and education-adjusted normative data [25]. For the present study, cognitive performance was summarized in three domains: attention, memory, and executive function. Attention was assessed using Digit Span Forward (DSF), Digit Span Backward (DSB), and the Korean Trail Making Test for the Elderly Part A (K-TMT-E Part A). Higher DSF and DSB scores indicated better performance, whereas shorter completion times on K-TMT-E Part A indicated better performance. Memory was assessed using the Seoul Verbal Learning Test (SVLT), including immediate recall total (sum across three trials) and delayed recall. Higher scores indicated better memory performance. Executive function was assessed using K-TMT-E Part B and the Controlled Oral Word Association Test (COWAT). Shorter completion times on K-TMT-E Part B and higher COWAT scores indicated better executive performance. Detailed descriptions of the cognitive measures and scoring are provided in Supplementary Table S1.

#### 2.3.2. Sociodemographic and Clinical Characteristics

Sociodemographic and clinical characteristics were obtained from self-report questionnaires and medical records. Sociodemographic variables included age, sex, and educational level. Clinical and health-related variables included body mass index (BMI), smoking status, alcohol use, HF phenotype, New York Heart Association (NYHA) functional class, Charlson Comorbidity Index (CCI), and functional status based on the Korean Activity Scale Index (KASI) classification [26]. HF phenotype was categorized on the basis of the left ventricular ejection fraction recorded at the time and retrospectively, according to contemporary guideline definitions [27] as heart failure with reduced (HFrEF), mildly reduced (HFmrEF), or preserved (HFpEF) ejection fraction.

#### 2.3.3. Anxiety and Depressive Symptoms

Anxiety symptoms were assessed using the Korean version of the Beck Anxiety Inventory (BAI), originally developed by Beck et al. [28] and translated and validated in Korean populations by Yook and Kim [29]. The BAI consists of 21 items rated on a 4-point scale (0–3), yielding a total score range of 0–63, with higher scores indicating greater anxiety severity. Internal consistency was Cronbach’s α = 0.86 in the Korean validation study [29] and 0.926 in the present sample. A total score of ≥8 was used to indicate clinically meaningful anxiety symptoms, consistent with the established severity ranges of the BAI [28,29].

Depressive symptoms were assessed using the Korean version of the Beck Depression Inventory-II (BDI-II), originally developed by Beck and Steer [30] and validated in Korean populations by Lim et al. [31]. The BDI-II comprises 21 items rated on a 4-point scale (0–3), with a total score range of 0–63; higher scores indicate more severe depressive symptoms. Internal consistency in the Korean validation study was reported as Cronbach’s α ≥ 0.94 [31], and Cronbach’s α was 0.903 in the present sample. A total score of ≥18 was used to indicate clinically meaningful depressive symptoms, corresponding to at least moderate severity on the BDI-II, based on the cut-offs adopted in the Korean validation study [30,31].

### 2.4. Data Collection

The primary dataset was prospectively collected between 16 December 2016 and 28 February 2018. For the original study, participants completed the self-report questionnaires and underwent standardized neuropsychological testing with the SNSB-II; the questionnaires were administered and scored by trained research personnel following standardized instructions, and the SNSB-II by qualified examiners using age- and education-adjusted normative data. Clinical characteristics, including HF phenotype and NYHA functional class, were abstracted from medical records using a standardized data collection form. Anxiety and depressive symptoms were treated as questionnaire-based symptom screens rather than as clinical diagnoses, and no separate diagnosis of anxiety or depression was made by a mental-health physician or the treating cardiologist. Prior to participation, all participants provided written informed consent covering these assessments and the research use of their clinical data.

### 2.5. Statistical Analysis

All analyses were conducted using IBM SPSS Statistics version 29.0 (IBM Corp., Armonk, NY, USA) and R version 4.3.2 (R Foundation for Statistical Computing, Vienna, Austria). No missing data were identified for the variables included in the final analyses; therefore, all participants were retained in the complete-case analytic sample. Descriptive statistics are presented as frequencies and percentages for categorical variables and as means and standard deviations for continuous variables.

Age- and education-adjusted norm-referenced SNSB-II scores were converted to z-scores in standard deviation units (z = [T − 50]/10) [25]. For the time-based subtests (K-TMT-E), scores were oriented so that, consistently with the other measures, lower z-scores indicate poorer performance, and reporting of standardized scores followed the American Academy of Clinical Neuropsychology recommendations [32]. Domain-specific cognitive impairment prevalence was estimated based on predefined impairment criteria applied within each cognitive domain. Specifically, impairment within a domain was defined as either at least two subtests with z ≤ −1.5 or at least one subtest with z ≤ −2.0. These actuarial criteria follow established neuropsychological approaches for defining domain-level impairment [33]. The prevalence of impairment was then summarized for each cognitive domain and for impairment in at least one domain.

Latent cognitive profiles were identified by latent profile analysis [34,35] using the domain composite z-scores for attention, memory, and executive function, each calculated as the mean of the corresponding age- and education-adjusted subtest z-scores. Because the K-TMT-E has a fixed maximum completion time, a small number of participants produced extreme standardized scores; to limit their disproportionate influence on the domain composites and the profile solution, standardized subtest scores exceeding ±3.29 SD—a conventional criterion for univariate outliers [36]—were winsorized to that threshold prior to analysis. Models were fitted in R (mclust, version 6.1.2) using a spherical (class-varying volume) parameterization applied consistently across two- to four-profile solutions, with multiple random starts to avoid local maxima. The number of profiles was determined using the AIC, BIC, sample-size-adjusted BIC, entropy, and a bootstrap likelihood-ratio test (BLRT) [37]; the three-profile solution was retained and interpreted as exploratory. Associations of profile membership with participant characteristics were examined using one-way analysis of variance or the chi-square (or Fisher’s exact) test; associations with anxiety and depressive symptoms were further examined using multinomial and Firth penalized logistic regression, with a sensitivity analysis based on pseudo-class draws to account for classification uncertainty [38]. The Benjamini–Hochberg false discovery rate correction was applied.

### 2.6. Ethical Considerations

The study was conducted in accordance with the Declaration of Helsinki and applicable local regulations. The original study protocol and the current secondary analysis were approved by the Institutional Review Board of Ajou University Hospital (IRB No. AJOUIRB-SUR-2016-431, approved on 16 December 2016). All participants received an explanation of the study purpose and procedures and provided written informed consent prior to their enrollment in the primary study. Participant data were collected prospectively for the primary study and were stored in an access-restricted database in accordance with institutional data management policies. All study records were managed using coded participant identifiers, and no direct personal identifiers were included in the analysis dataset.

## 3. Results

### 3.1. Sociodemographic and Clinical Characteristics of Participants

A total of 106 patients with HF were included in the analysis. The mean age was 65.93 years (SD = 10.56), and 70.8% were male. HF phenotypes were evenly distributed (HFrEF, 39.6%; HFmrEF, 20.8%; and HFpEF, 39.6%), and most patients were NYHA functional class I–II (93.4%). Clinically meaningful anxiety (BAI ≥ 8) and depressive (BDI-II ≥ 18) symptoms were present in 64.2% and 27.4% of participants, respectively. Full sociodemographic and clinical characteristics are provided in Table 1.

### 3.2. Prevalence and Patterns of Cognitive Impairment

Raw subtest performance and norm-referenced z-scores are summarized in Table 2. Across domains, mean z-scores were generally close to the normative mean for attention (e.g., DSF, mean = 0.28, SD = 1.06; K-TMT-E Part A, mean = 0.08, SD = 1.01), whereas lower mean performance was observed for memory (SVLT immediate recall, mean = −0.43, SD = 1.00; SVLT delayed recall, mean = −0.60, SD = 1.01). Executive function subtests also showed modestly lower mean performance overall (e.g., K-TMT-E Part B, mean = −0.01, SD = 1.21). Based on the predefined domain-level impairment criteria, impairment was identified in 11.3% of participants for attention, 15.1% for memory, and 17.9% for executive function. Overall, cognitive impairment in at least one domain was present in 34 participants (32.1%).

### 3.3. Model Selection for LPA

LPA was conducted using domain composite z-scores for attention, memory, and executive function. The information criteria did not converge on a single solution: the AIC and sample-size-adjusted BIC continued to decrease toward the four-profile solution, whereas the BIC favored the two-profile solution (Supplementary Table S2). To adjudicate between solutions, a bootstrap likelihood-ratio test (BLRT) was applied; it significantly favored the three-profile over the two-profile solution (*p* = 0.005) but did not favor the four-profile over the three-profile solution (*p* = 1.00). The four-profile solution additionally produced a spurious class of only three participants. The three-profile solution was therefore retained, supported by the BLRT and by balanced class sizes (*n* = 48, 32, and 26) with acceptable classification quality (entropy = 0.63; Supplementary Table S3).

### 3.4. Domain Composite Profiles by Latent Class

**Figure 1:**
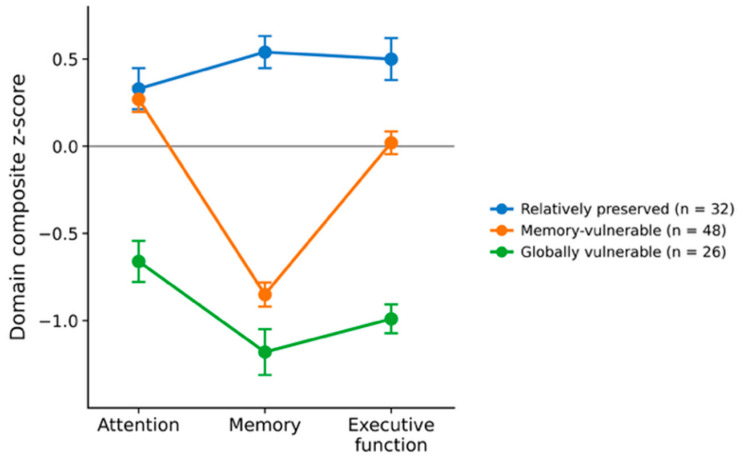
Latent cognitive profiles based on domain composite z-scores. Values represent mean domain composite z-scores for attention, memory, and executive function across the three latent profiles. Error bars indicate standard errors. A value of 0 indicates the normative mean, and lower values indicate poorer performance.

The final three-profile solution comprised 32 relatively preserved participants (30.2%), 48 memory-vulnerable participants (45.3%), and 26 globally vulnerable participants (24.5%) (Table 3; Figure 1). The relatively preserved profile showed mean z-scores at or above the normative mean across domains (attention, mean = 0.33, SD = 0.67; memory, mean = 0.54, SD = 0.52; executive function, mean = 0.50, SD = 0.68). The memory-vulnerable profile was characterized by selectively lower memory performance (mean = −0.85, SD = 0.48), whereas attention (mean = 0.27, SD = 0.51) and executive function (mean = 0.02, SD = 0.45) remained close to the normative mean. The globally vulnerable profile showed low mean performance across all domains, with the most pronounced deficit observed in memory (mean = −1.18, SD = 0.67), followed by executive function (mean = −0.99, SD = 0.42) and attention (mean = −0.66, SD = 0.60). Subtest-level z-scores for each profile are provided in Supplementary Table S4.

### 3.5. Associations of Participant Characteristics with Latent Cognitive Profile Membership

In the multinomial models, clinically meaningful anxiety symptoms were associated with cognitive vulnerability, whereas depressive symptoms were not (Table 4). In multinomial models (reference, relatively preserved), anxiety was associated with higher odds of membership in the memory-vulnerable profile (OR = 2.81, 95% CI: 1.03–7.64, *p* = 0.044) and the globally vulnerable profile (OR = 4.71, 95% CI: 1.31–16.93, *p* = 0.018), and in a Firth penalized model, with membership in any vulnerable profile (OR = 3.39, 95% CI: 1.46–8.11, *p* = 0.005). Depressive symptoms were not significantly associated with either profile (both *p* > 0.55). However, in a sensitivity analysis that accounted for classification uncertainty using pseudo-class draws, these associations were attenuated and no longer statistically significant (memory-vulnerable, anxiety OR = 2.21, 95% CI: 0.67–7.25, *p* = 0.19; globally vulnerable, anxiety OR = 2.94, 95% CI: 0.75–11.54, *p* = 0.122). When all candidate characteristics (*n* = 15) were screened univariately, only anxiety showed a nominal association (*p* = 0.009), which did not survive Benjamini–Hochberg FDR correction (FDR-adjusted *p* = 0.14). No sociodemographic or routine clinical characteristic was significantly associated with profile membership. These associations should therefore be regarded as exploratory.

## 4. Discussion

In this secondary analysis, we characterized cognitive vulnerability in outpatients with HF from two complementary perspectives: domain-specific impairment prevalence and domain-level performance patterns identified by LPA. Cognitive impairment in at least one domain was present in approximately one-third of participants, most frequently in executive function and memory. Moving beyond the overall prevalence of impairment, LPA indicated that cognitive vulnerability was heterogeneous in pattern, which may be clinically relevant because the functional consequences of cognitive dysfunction in HF are likely to depend on how weaknesses are distributed within individuals rather than on the mere presence of impairment [12,39,40]. The overall prevalence fell within the broad range reported previously [5,6]. While this variability is usually attributed to methodological and sampling differences [5,11], our identification of distinct profiles within a single outpatient sample suggests that part of this variability may also reflect genuine heterogeneity within the HF population, which prevalence estimates alone can obscure [11,12]. Brief cognitive screening can identify patients who may require further evaluation but is less suited to characterizing the structure of vulnerability or anticipating specific functional difficulties [11]. Given that HF self-care is cognitively complex [12,41], two patients with a similar overall burden may nonetheless face different self-management barriers if their vulnerability is concentrated in different domains.

Three profiles were identified: relatively preserved, memory-vulnerable, and globally vulnerable. The memory-vulnerable profile was the largest subgroup, suggesting that selective memory vulnerability may be a particularly common form of cognitive difficulty in HF outpatients, consistent with reports of frequent immediate- and delayed-memory deficits even when other domains vary [15,16,42]. As self-care requires patients not only to understand information during a clinic visit but also to retain and retrieve it for medication adherence, symptom monitoring, and action planning [41,43], our findings conceptually align with the need to tailor education to reduce memory burden. Specifically, prior literature suggests that approaches such as limiting education to a small number of core messages, providing written summaries and visual materials, and using simple action plans may be especially relevant for patients with this type of cognitive profile [12,43,44].

The globally vulnerable profile, with low performance across all domains including executive function, may represent a more severe form of vulnerability. HF self-care requires more than remembering isolated instructions; it also depends on prioritizing symptoms, organizing behaviors, inhibiting ineffective responses, and adapting plans as symptoms or treatment demands change—functions supported by executive processes [12,13,41]. When executive dysfunction co-occurs with broader cognitive vulnerability, the resulting functional burden may extend beyond memory-related difficulty alone. Therefore, it is hypothesized that such patients may benefit from more structured, stepwise support—such as simplified self-care goals, explicit task sequencing, standardized tools like checklists or monitoring logs, and where appropriate, caregiver involvement—rather than repetition of educational content alone [12,13]. Conversely, the presence of the relatively preserved profile demonstrates that cognitive vulnerability is not inevitable even among clinically similar HF outpatients, confirming that cognitive performance varies in both degree and pattern [12,16].

Among the characteristics examined, only anxiety symptoms were associated with cognitive vulnerability, whereas depressive symptoms were not. Compared to the relatively preserved profile, anxiety was associated with higher odds of memory-vulnerable and any-vulnerable membership; however, the globally vulnerable contrast was attenuated when accounting for classification uncertainty, and no association survived FDR correction. These associations should therefore be regarded as exploratory and hypothesis-generating, given the modest sample and the number of comparisons. When present, anxiety may interfere with the attentional and executive resources needed for interpreting symptoms and enacting self-care, and it has been linked to symptom uncertainty, hypervigilance, and poorer outcomes in HF [45,46]. This pattern is consistent with a biopsychosocial view in which emotional symptoms form part of the context in which cognitive vulnerability is expressed [12,13,39].

Depressive symptoms were not consistently linked to any profile once overfitting was addressed [39,45,47]. The relationship between emotional and cardiac symptoms may also be bidirectional: rather than acting solely as antecedent risk factors, anxiety and depressive symptoms may partly reflect the somatic, inflammatory, and hemodynamic burden of HF and thus form part of its clinical expression [13,39,45]. Furthermore, given the cross-sectional design, directionality cannot be determined. Additionally, the null associations observed for sociodemographic and clinical indicators should be interpreted cautiously rather than as evidence of unimportance [8,12,13]. These null findings may reflect limited statistical power or the possibility that emotional symptoms are more salient correlates than the conventional indicators examined here.

Clinically, the profiles should be viewed not as diagnostic categories but as a practical stratification that may help guide the intensity and delivery of education, monitoring, and support according to a patient’s cognitive limitations [12]. Cognitive assessment may be most informative when interpreted alongside emotional symptoms, particularly when self-management difficulties appear disproportionate to conventional clinical indicators [8,12,13]. To build on these exploratory findings, future work should examine the stability, reproducibility, and prognostic relevance of these profiles in larger, multicenter cohorts, specifically investigating their links to self-care, readmission, and mortality [3,12,16,37,39].

Several limitations should be considered. First, the cross-sectional design precludes causal inference regarding cognitive vulnerability, emotional symptoms, and clinical characteristics. Second, because multiple variables were compared, type I errors cannot be excluded. Third, as the dataset was fixed, no power analysis was possible for the LPA. Consequently, the identified profiles and their correlates remain exploratory; their enumeration may have been influenced by sample size and model specification, necessitating validation in larger samples. Furthermore, the dataset was collected between 2016 and 2018. Although guideline-directed therapies have since evolved, the observed prevalence and cognitive structures likely reflect relatively stable neurocognitive characteristics rather than acute treatment responses. Additionally, the exclusion of patients with stroke, dementia, or centrally acting medications makes the prevalence a conservative estimate, while recruitment from a single tertiary clinic with a requirement to complete testing limits generalizability to inpatients or more severely impaired patients. Anxiety and depression were assessed as self-reported symptom burden rather than clinical diagnoses, and the dataset did not capture subsequent referral or management. Finally, the visuospatial domain was not included and thus not represented in the profiles, and the domains comprised unequal numbers of subtests, which may have differentially affected the precision of the composite z-scores.

## 5. Conclusions

In Korean outpatients with HF, cognitive impairment was identified in approximately one-third of patients, with executive function and memory emerging as the most frequently affected domains. Three latent cognitive profiles were observed—relatively preserved, memory-vulnerable, and globally vulnerable. In exploratory analyses, anxiety symptoms showed a possible association with cognitive vulnerability, whereas depressive symptoms showed no significant association; however, no association remained statistically significant after correction for multiple comparisons, and these results should therefore be regarded as hypothesis-generating. These findings support the view that cognitive vulnerability in HF is heterogeneous and may not be adequately captured by overall prevalence estimates or brief screening alone. Given the exploratory design and modest sample size, consideration of both domain-specific and profile-level cognitive patterns may help generate hypotheses for more individualized outpatient assessment and support, which require validation in larger, multicenter, longitudinal cohorts before clinical application.

## Figures and Tables

**Table 1:** Sociodemographic and clinical characteristics of the participants (*N* = 106).

Characteristics	Categories	*n* (%) or Mean ± SD
Age (years)		65.93 ± 10.56
	45–54	20 (18.9)
	55–64	33 (31.1)
	≥65	53 (50.0)
Sex	Male	75 (70.8)
	Female	31 (29.2)
Educational level	Elementary school	25 (23.6)
	Middle school	13 (12.2)
	High school	43 (40.6)
	University or higher	25 (23.6)
Body mass index (kg/m^2^)		24.89 ± 3.70
	Normal (<23)	30 (28.4)
	Overweight (23–24.9)	38 (35.8)
	Obese (≥25)	38 (35.8)
Smoking status	None	61 (57.5)
	Former smoker	37 (35.0)
	Current smoker	8 (7.5)
Alcohol use	None	54 (51.0)
	Former drinker	28 (26.4)
	Current drinker	24 (22.6)
Heart failure phenotype	HFrEF	42 (39.6)
	HFmrEF	22 (20.8)
	HFpEF	42 (39.6)
New York Heart Association class	I	56 (52.8)
	II	43 (40.6)
	III	7 (6.6)
Comorbidity		0.78 ± 1.00
	0	51 (48.1)
	1	39 (36.8)
	≥2	16 (15.1)
Korean Activity Scale Index		46.28 ± 20.97
	Class 1	53 (50.0)
	Class 2	33 (31.1)
	Class 3	20 (18.9)
Anxiety symptoms		11.74 ± 8.82
	None (0–7)	38 (35.8)
	Significant (≥8)	68 (64.2)
Depressive symptoms		13.27 ± 8.71
	None (0–17)	77 (72.6)
	Significant (≥18)	29 (27.4)

Note. Data are *n* (%) or mean ± SD. Clinically meaningful anxiety and depressive symptoms were defined using validated cut-offs (BAI ≥ 8; BDI-II ≥ 18). Abbreviations: BAI, Beck Anxiety Inventory; BDI-II, Beck Depression Inventory-II; HFmrEF, heart failure with mildly reduced ejection fraction; HFpEF, heart failure with preserved ejection fraction; HFrEF, heart failure with reduced ejection fraction; SD, standard deviation.

**Table 2:** Cognitive subtest performance and domain-specific cognitive impairment prevalence (*N* = 106).

Domain	Test	Raw Mean ± SD	z	z ≤ −1.5	z ≤ −2.0	Domain Impaired
			Mean ± SD	*n* (%)	*n* (%)	*n* (%)
Attention	DSF	6.31 ± 1.57	0.28 ± 1.06	5 (4.7)	3 (2.8)	12 (11.3)
	DSB	3.78 ± 1.29	−0.19 ± 1.09	5 (4.7)	4 (3.8)	
	K-TMT-E Part A (sec)	28.19 ± 18.33	0.08 ± 1.01	8 (7.5)	7 (6.6)	
Memory	SVLT Immediate recall	18.44 ± 4.60	−0.43 ± 1.00	13 (12.3)	5 (4.7)	16 (15.1)
	SVLT Delayed recall	5.10 ± 2.62	−0.60 ± 1.01	17 (16.0)	12 (11.3)	
Executive function	K-TMT-E Part B (sec)	65.73 ± 70.88	−0.01 ± 1.21	12 (11.3)	11 (10.4)	19 (17.9)
	COWAT Category: Animal	15.39 ± 4.78	−0.05 ± 1.12	9 (8.5)	4 (3.8)	
	COWAT Category: Supermarket	16.06 ± 6.32	−0.24 ± 1.09	10 (9.4)	4 (3.8)	
	COWAT Phonemic total	25.08 ± 10.68	−0.03 ± 1.08	13 (12.3)	3 (2.8)	

Values are presented as mean ± SD. Lower z-scores indicate poorer performance. Abbreviations: COWAT, Controlled Oral Word Association Test; DSB, Digit Span Backward; DSF, Digit Span Forward; K-TMT-E, Korean Trail Making Test for the Elderly; SVLT, Seoul Verbal Learning Test.

**Table 3:** Domain composite z-score summaries across latent cognitive profiles (*N* = 106).

Variable	Relatively Preserved (*n* = 32)	Memory-Vulnerable (*n* = 48)	Globally Vulnerable (*n* = 26)
	Mean ± SD	Mean ± SD	Mean ± SD
Attention	0.33 ± 0.67	0.27 ± 0.51	−0.66 ± 0.60
Memory	0.54 ± 0.52	−0.85 ± 0.48	−1.18 ± 0.67
Executive function	0.50 ± 0.68	0.02 ± 0.45	−0.99 ± 0.42

**Table 4:** Associations of anxiety and depressive symptoms with cognitive profile membership (multinomial, Firth-penalized, and classification-uncertainty models).

Model/Contrast	Predictor	OR (95% CI)	*p*
Multinomial (ref. relatively preserved)			
Memory-vulnerable	Anxiety symptoms (BAI ≥ 8)	2.81 (1.03–7.64)	0.044
	Depressive symptoms (BDI-II ≥ 18)	1.03 (0.31–3.39)	0.967
Globally vulnerable	Anxiety symptoms (BAI ≥ 8)	4.71 (1.31–16.93)	0.018
	Depressive symptoms (BDI-II ≥ 18)	1.48 (0.40–5.40)	0.556
Firth-penalized (ref. relatively preserved)			
Any vulnerable	Anxiety symptoms (BAI ≥ 8)	3.39 (1.46–8.11)	0.005
	Depressive symptoms (BDI-II ≥ 18)	1.86 (0.72–5.34)	0.204
Pseudo-class (classification uncertainty)			
Memory-vulnerable	Anxiety symptoms (BAI ≥ 8)	2.21 (0.67–7.25)	0.190
	Depressive symptoms (BDI-II ≥ 18)	1.23 (0.33–4.62)	0.757
Globally vulnerable	Anxiety symptoms (BAI ≥ 8)	2.94 (0.75–11.54)	0.122
	Depressive symptoms (BDI-II ≥ 18)	2.05 (0.52–8.04)	0.302

Note. Estimates are exploratory. Multinomial models used the relatively preserved profile as the reference category, and the Firth penalized model estimated the odds of membership in any vulnerable profile. A classification-uncertainty analysis using pseudo-class draws is reported as a sensitivity analysis. After Benjamini–Hochberg FDR correction across all candidate characteristics (*n* = 15), no variable remained significantly associated with profile membership (anxiety, FDR-adjusted *p* = 0.14). Abbreviations: CI, confidence interval; FDR, false discovery rate; OR, odds ratio; ref, reference group.

## Data Availability

The data contain potentially identifying and sensitive patient information and therefore cannot be shared publicly. De-identified minimal datasets and the codebook necessary to reproduce the findings are available from the Institutional Review Board of Ajou University Hospital (AJOUIRB) for researchers who meet the criteria for access to confidential data. Requests for access should be submitted to the IRB through the institution’s official contact channel.

## Supplementary Materials

The following supporting information can be downloaded at: https://www.mdpi.com/article/10.3390/jcm15155888/s1, Supplementary Table S1: Measures used in the present analysis (cognitive domains and subtests [SNSB-II–based] and self-report symptom and functional-status questionnaires); Supplementary Table S2: Model fit indices and classification quality for latent profile analysis solutions (2–4 profiles); Supplementary Table S3: Average latent-class posterior probabilities by most likely profile; Supplementary Table S4: Subtest z-scores (mean ± SD) by latent cognitive profile.

## Abbreviations

The following abbreviations are used in this manuscript: AIC, Akaike information criterion; BAI, Beck Anxiety Inventory; BDI-II, Beck Depression Inventory-II; BIC, Bayesian information criterion; BMI, body mass index; CCI, Charlson Comorbidity Index; CI, confidence interval; COWAT, Controlled Oral Word Association Test; DSB, Digit Span Backward; DSF, Digit Span Forward; FDR, false discovery rate; HF, heart failure; HFmrEF, heart failure with mildly reduced ejection fraction; HFpEF, heart failure with preserved ejection fraction; HFrEF, heart failure with reduced ejection fraction; KASI, Korean Activity Scale Index; K-TMT-E, Korean Trail Making Test for the Elderly; LPA, latent profile analysis; NYHA, New York Heart Association; OR, odds ratio; SD, standard deviation; SNSB-II, Seoul Neuropsychological Screening Battery-II; STROBE, Strengthening the Reporting of Observational Studies in Epidemiology; SVLT, Seoul Verbal Learning Test.
