# Supplementary material for: Prevalence and Latent Profiles of Cognitive Impairment in Patients with Heart Failure: A Secondary Data Analysis
Source: J Clin Med. 2026 Jul 28;15(15):5888. doi: 10.3390/jcm15155888 (PMC13466635; doi:10.3390/jcm15155888)
Supplement: Supplementary file 1 [file jcm-15-05888-s001.zip › jcm-4415087-supplementary.pdf]

Supplementary Materials

Supplementary Table S1. Measures used in the present analysis (cognitive domains and subtests [SNSB-II–based] and self-report symptom and functional-status questionnaires).

(A) Cognitive domains and subtests (SNSB-II–based)

Cognitive domain	Operational definition (this study)	Subtests (SNSB-II)	Procedures / scoring
Attention	Performance reflecting sustained attention together with visual search and processing speed.	Digit Span Forward (DSF); Digit Span Backward (DSB); Korean Trail Making Test for the Elderly, Part A (K-TMT-E A)	For DSF/DSB, the examiner presented sequences of digits and participants immediately repeated them in the same (forward) or reverse (backward) order; higher scores indicated better performance. For K-TMT-E Part A, participants connected numbers in ascending order as quickly and accurately as possible; completion time (seconds) was recorded, with shorter time indicating better performance.
Memory	Verbal learning and episodic recall.	Seoul Verbal Learning Test (SVLT) immediate recall total; SVLT delayed recall	A 12-word list was administered, and immediate free recall was repeated across three trials to compute the immediate recall total; a delayed recall trial followed after a retention interval. Immediate recall total was the sum of correctly recalled words across three trials, and delayed recall was the number of words recalled after the retention interval; higher scores indicated better memory performance.
Executive function	Executive processes involving set-shifting and organization, and verbal fluency.	K-TMT-E Part B (K-TMT-E B); Controlled Oral Word Association Test (COWAT): category fluency (animal, supermarket) and phonemic fluency total	For K-TMT-E Part B, participants alternated between numbers and Korean letters while connecting stimuli in sequence; completion time (seconds) was recorded, with shorter time indicating better executive performance. For COWAT, the number of admissible words generated within the fixed time limit was summed for each condition; higher scores indicated better performance.

Note. SNSB-II, Seoul Neuropsychological Screening Battery-II. Age- and education-adjusted normative z-scores were used for all subtests. The visuospatial domain available in the SNSB-II was not included

because the present analysis focused on the attention, memory, and executive function domains most relevant to heart-failure self-management.

(B) Self-report symptom and functional-status questionnaires

**Instrument**	**Construct / structure**	**Scoring & cutoff**	**Source / Korean validation**
BAI (Beck Anxiety Inventory), Korean version	Anxiety symptom severity; 21 items, 4-point scale (0–3)	Total 0–63; higher = greater severity. Clinically meaningful anxiety: total ≥ 8. Present-sample Cronbach's α = 0.926	Beck et al., 1988; Korean validation: Yook & Kim, 1997
BDI-II (Beck Depression Inventory-II), Korean version	Depressive symptom severity; 21 items, 4-point scale (0–3)	Total 0–63; higher = more severe. Clinically meaningful depression: total ≥ 18 (≥ moderate). Present-sample Cronbach's α = 0.903	Beck & Steer, 1996; Korean validation: Lim et al., 2011
KASI (Korean Activity Scale/Index)	Functional/physical activity status	Weighted activity items; higher = better functional capacity	Sung et al., 2000
SNSB-II (Seoul Neuropsychological Screening Battery-II)	Standardized neuropsychological battery (attention, memory, executive function)	Age- and education-adjusted norm-referenced scores converted to z-scores. Domain impairment: ≥2 subtests z ≤ −1.5 or ≥1 subtest z ≤ −2.0	Ryu & Yang, 2023 (subtest details in Supplementary Table S1)

Note. The Beck Anxiety Inventory and Beck Depression Inventory-II are copyrighted instruments; accordingly, only their structure, scoring, and validated cutoffs are summarized here, and the full item sets are not reproduced. Korean validation sources are listed in the final column. Abbreviations: BAI, Beck Anxiety Inventory; BDI-II, Beck Depression Inventory-II; KASI, Korean Activity Scale Index; SNSB-II, Seoul Neuropsychological Screening Battery-II.

Supplementary Table S2. Model fit indices and classification quality for latent profile analysis solutions (2–4 profiles)**.**

No. of profiles	Log-likelihood	Free parameters	AIC	BIC	SABIC	Entropy	Class sizes (n)	Smallest class (n)
2	−356.53	9	731.05	755.02	726.59	0.585	62, 44	44
3	−346.24	14	720.48	757.77	713.54	0.633	48, 32, 26	26
4	−338.95	19	715.91	766.51	706.48	0.719	51, 27, 25, 3	3

A bootstrap likelihood-ratio test favored the three-profile solution (two vs. three profiles, p = 0.005; three vs. four profiles, p = 1.00).

Note. The three-profile solution was selected as the final, exploratory solution. The information criteria did not converge on a single solution: the AIC and SABIC were lowest for the four-profile solution, whereas the BIC was lowest for the two-profile solution. The four-profile solution was not retained because it produced a very small class (n = 3). The three-profile solution was retained on the basis of the bootstrap likelihood-ratio test (BLRT), which favored three over two profiles (p = 0.005) but not four over three profiles (p = 1.00), together with the absence of a very small class, balanced and clinically interpretable class sizes, and acceptable classification quality. AIC, Akaike information criterion; BIC, Bayesian information criterion (lower values indicate better fit); SABIC, sample-size-adjusted Bayesian information criterion. Entropy ranges from 0 to 1, with higher values indicating clearer classification.

Supplementary Table S3. Average latent-class posterior probabilities by most likely profile.

Most likely profile	Relatively preserved	Memory-vulnerable	Globally vulnerable	n
Relatively preserved	0.890	0.105	0.005	32
Memory-vulnerable	0.122	0.775	0.102	48
Globally vulnerable	0.012	0.118	0.870	26

Rows group cases by their most likely profile; columns give the mean posterior probability of each profile. Diagonal values indicate classification certainty. Overall entropy = 0.633.

Supplementary Table S4. Subtest z-scores (mean ± SD) by latent cognitive profile.

Subtest	Relatively preserved (n = 32)	Memory-vulnerable (n = 48)	Globally vulnerable (n = 26)
DSF	0.32 ± 1.07	0.61 ± 0.91	−0.38 ± 1.03
DSB	0.18 ± 1.19	−0.01 ± 0.92	−0.95 ± 0.93
K-TMT-E Part A	0.47 ± 0.71	0.22 ± 0.79	−0.66 ± 1.29
SVLT immediate recall	0.58 ± 0.74	−0.73 ± 0.54	−1.12 ± 1.02
SVLT delayed recall	0.49 ± 0.58	−0.98 ± 0.74	−1.24 ± 0.79
K-TMT-E Part B	0.44 ± 0.85	0.21 ± 0.87	−0.97 ± 1.60
COWAT category: animal	0.60 ± 1.02	0.05 ± 0.98	−1.02 ± 0.82
COWAT category: supermarket	0.44 ± 1.07	−0.16 ± 0.89	−1.22 ± 0.66
COWAT phonemic total	0.51 ± 1.16	0.00 ± 0.85	−0.74 ± 1.00

Values are winsorized age- and education-adjusted z-scores. Abbreviations: COWAT, Controlled Oral Word Association Test; DSB, Digit Span Backward; DSF, Digit Span Forward; K-TMT-E, Korean Trail Making Test for the Elderly; SVLT, Seoul Verbal Learning Test.
